# When Clinical Judgment Goes Beyond Balance, Eyes, Face, Arm, Speech (BEFAST): Acute Confusion Prompting Stroke Code Activation in Dominant Hemisphere Large Vessel Occlusion

**DOI:** 10.7759/cureus.113578

**Published:** 2026-07-29

**Authors:** Humaid Sadiq, Abdulrahman Qasem, Mansoor M Husain, Jamal Alkoteesh

**Affiliations:** 1 Emergency Medicine, Tawam Hospital, Al Ain, ARE; 2 Interventional Radiology, Tawam Hospital, Al Ain, ARE

**Keywords:** acute confusion, acute ischemic stroke (ais), befast, emergency department stroke recognition, internal carotid artery occlusion, large vessel occlusion, mechanical thrombectomy, stroke chameleons

## Abstract

Acute ischemic stroke can present with atypical features, including confusion, disorientation, or other cortical manifestations that may not be captured by commonly used stroke recognition tools such as Balance, Eyes, Face, Arm, Speech (BEFAST), posing a diagnostic challenge in the emergency department and potentially delaying identification of large vessel occlusion (LVO) and reperfusion therapy. We report a 46-year-old previously independent male who presented with acute-onset confusion following a syncopal episode that was preceded by headache and dizziness. Initial BEFAST assessment was unremarkable with preserved motor function, but a stroke code was activated due to acute cognitive change. Neuroimaging demonstrated early ischemic changes in the left cerebral hemisphere with complete occlusion of the terminal segment of the left internal carotid artery (ICA). The patient was not eligible for intravenous thrombolysis and subsequently underwent successful mechanical thrombectomy with complete reperfusion. His clinical course was notable for persistent aphasia with preserved motor strength, later re-occlusion of the ICA managed conservatively, and gradual improvement with supportive care and speech therapy. This case highlights acute confusion as a potential presenting feature of ischemic stroke due to LVO and emphasizes that reliance solely on initial rapid stroke screening tools may miss patients with predominantly cortical involvement, underscoring the importance of early clinical judgment-driven stroke activation and prompt neuroimaging in atypical presentations.

## Introduction

Sometimes, confusion is the first clue. Acute ischemic stroke, caused by sudden interruption of cerebral blood flow (CBF) due to arterial occlusion, accounts for approximately 85% of all stroke cases worldwide [1]. It can range from classic motor and speech deficits to subtle cognitive or behavioural changes [1], making early recognition a challenge in the emergency setting. While stroke recognition tools such as Balance, Eyes, Face, Arm, Speech (BEFAST) [2] emphasize focal motor, speech, and visual symptoms, they do not account for acute confusion or disorientation, which may be easily attributed to non-neurological causes in the emergency department (ED) and may delay diagnosis if not appropriately recognized [3]. Delayed recognition of acute ischemic stroke may result in missed opportunities for reperfusion therapy, larger infarct size, increased disability, and poorer functional outcomes [1].

Large vessel occlusion (LVO), particularly involving the internal carotid artery (ICA), may manifest with predominant cortical dysfunction, including impaired orientation, language disturbance, and higher cognitive deficits, even in the absence of early motor weakness [4,5]. In the ED, such presentations pose a diagnostic challenge, as acute confusion is frequently attributed to metabolic, toxic, or systemic causes rather than acute cerebrovascular disease [3].

Early activation of a stroke code based on clinical suspicion remains essential, as timely neuroimaging can reveal otherwise unsuspected large vessel pathology and guide reperfusion strategies [1]. Mechanical thrombectomy has become the standard of care for selected patients with LVO, with angiographic success commonly graded using the Thrombolysis in Cerebral Infarction (TICI) scale [6], where higher grades correlate with improved clinical outcomes [1,4].

We report a case of terminal ICA occlusion in which stroke code activation was driven primarily by acute confusion and disorientation, despite an initially unremarkable BEFAST assessment. This case underscores the importance of maintaining a high index of suspicion for acute ischemic stroke in patients presenting with acute cognitive symptoms and highlights key considerations in diagnostic evaluation and management.

## Case presentation

Initial presentation

A 46-year-old previously independent male presented to the ED approximately five hours after the onset of acute confusion following a syncopal episode. The event was preceded by severe headache and dizziness. There was no history of illicit drug use or other known medical comorbidities. During the initial triage assessment, stroke code activation was not considered, as the rapid BEFAST assessment was unremarkable. On subsequent reassessment in the resuscitation area, the patient remained awake but disoriented to time and place. Repeat bedside assessment demonstrated no obvious facial asymmetry, limb weakness, dysarthria, or gross visual deficits, and the BEFAST assessment remained unremarkable. The patient was afebrile, with no clinical evidence of neck stiffness. The initial differential diagnoses included acute ischemic stroke, postictal confusion following an unwitnessed seizure, and metabolic encephalopathy. However, the abrupt onset of persistent disorientation prompted the emergency physician to activate the stroke code approximately 10 minutes after ED arrival based on clinical judgment, despite the absence of obvious focal neurological deficits during the initial rapid assessments. Non-contrast CT was performed within the subsequent 10 minutes, followed by CT perfusion imaging approximately 15 minutes later.

Initial imaging and early radiological findings

**Figure 1:**
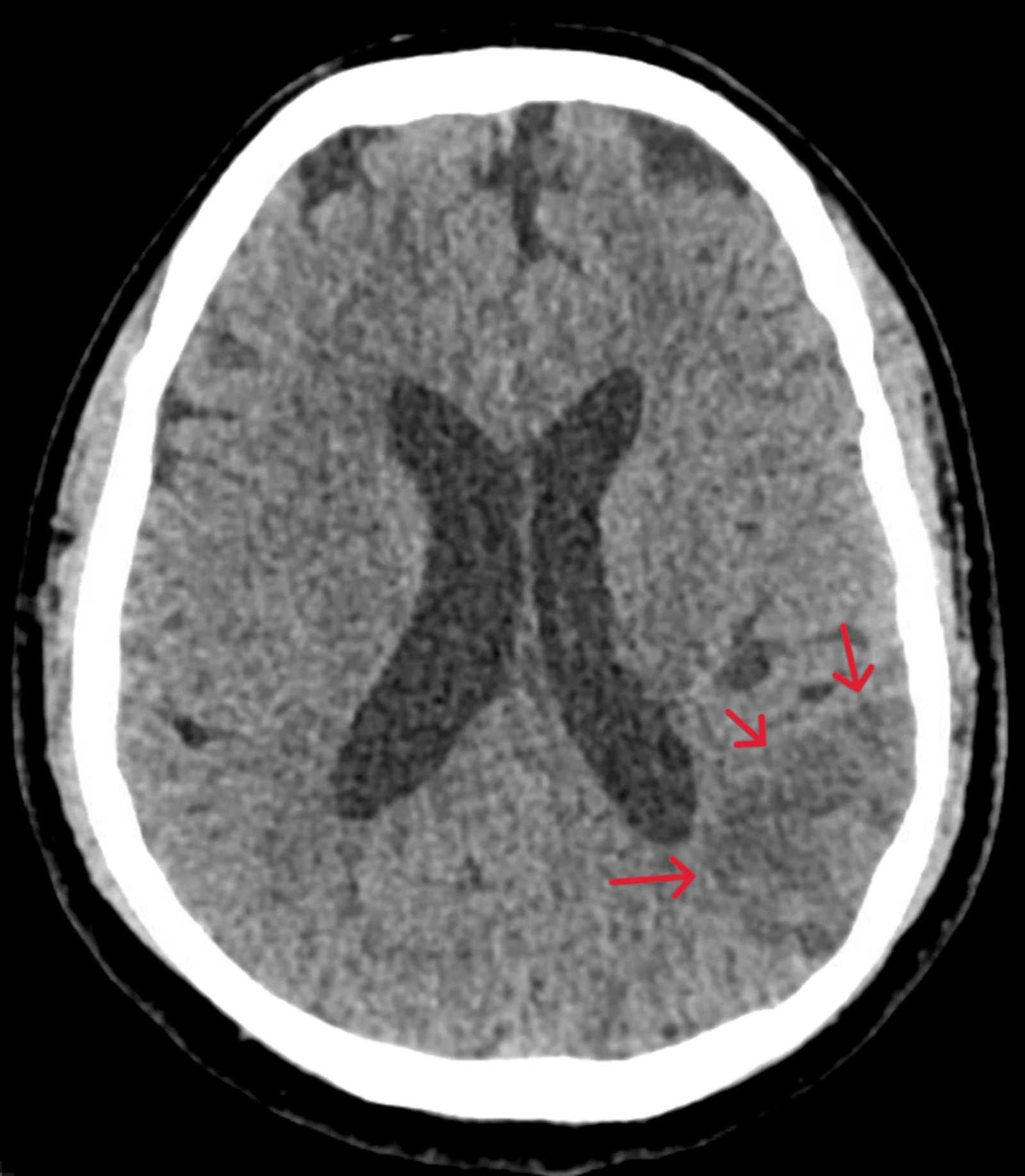
Non-contrast computed tomography (CT) brain showing established hypodensity in the left parieto-occipital region, consistent with acute infarction (as indicated by the red arrows).

A non-contrast computed tomography (CT) scan of the brain demonstrated no acute intracranial hemorrhage, with an Alberta Stroke Program Early CT Score (ASPECTS) [7] of 8, suggesting only early ischemic changes. Established hypodensities were also noted in the left parieto-occipital region, with additional involvement of the posterior and deep white matter of the left frontal lobe (Figure 1). No frank intracranial hemorrhage was identified, although small cortical linear densities suggestive of petechial hemorrhage were noted.

Neurological examination and stroke severity assessment

Following neuroimaging suggestive of acute ischemic stroke, a formal neurological examination was performed, revealing sensory aphasia, a right visual field deficit, and abnormal finger-to-nose testing, without facial asymmetry or clear limb motor weakness. Based on these findings, the patient's National Institutes of Health Stroke Scale (NIHSS) [8] score was calculated as 7. Subsequent formal neurological assessment was consistent with sensory aphasia with features of transcortical (innominate) aphasia.

Initial laboratory investigations

Initial laboratory investigations demonstrated a normal complete blood count, normal serum electrolyte levels, preserved renal function, and normal liver function tests. High-sensitivity cardiac troponin I was negative (Table 1), and electrocardiography (ECG) showed no acute abnormalities. Initial venous blood gas analysis was unremarkable except for hyperglycemia (19.0 mmol/L), consistent with the serum glucose level of 18.7 mmol/L (Table 2). Although hyperglycemia was identified, the absence of electrolyte abnormalities, renal dysfunction, or other metabolic derangements made acute metabolic encephalopathy an unlikely explanation for the patient's presentation.

**Table 1:** Initial laboratory investigations done in the emergency department (ED). eGFR: estimated glomerular filtration rate; AST: aspartate aminotransferase; ALT: alanine aminotransferase; ALP: alkaline phosphatase

Test	Result	Reference range	Unit
Sodium (Na)	135	136-145	mmol/L
Potassium (K)	4.2	3.5-5.1	mmol/L
Chloride (Cl)	102	98-107	mmol/L
Creatinine	66.4	53.0-114.9	micromol/L
eGFR	127	>=60	mL/min
AST	20	11-34	IU/L
ALT	17	<=45	IU/L
ALP	89	50-116	IU/L
Troponin-I	<10.00	<=34.20	ng/L
Glucose (Glu)	18.7	3.9-7.8	mmol/L

**Table 2:** Initial venous blood gas (VBG) results.

Test	Result	Reference range	Unit
pH	7.36	7.35-7.45	-
pCO_2_	49.9	35.0-45.0	mmHg
pO_2_	28.8	25.0-40.0	mmHg
HCO_3_	28	22-26	mmol/L
Base excess (BE)	1.4	-2.0 to 2.0	mmol/L
Total hemoglobin (THB)	150	120-170	g/L
Carboxyhemoglobin (COHb)	0.0	0.0-2.0	%
Potassium (K)	4.3	3.4-5.1	mmol/L
Chloride (Cl)	100	98-107	mmol/L
Sodium (Na)	136	136-145	mmol/L
Glucose (Glu)	19.0	3.9-6.0	mmol/L
Lactate (Lac)	1.8	0.5-2.2	mmol/L

Vascular imaging and perfusion analysis

**Figure 2:**
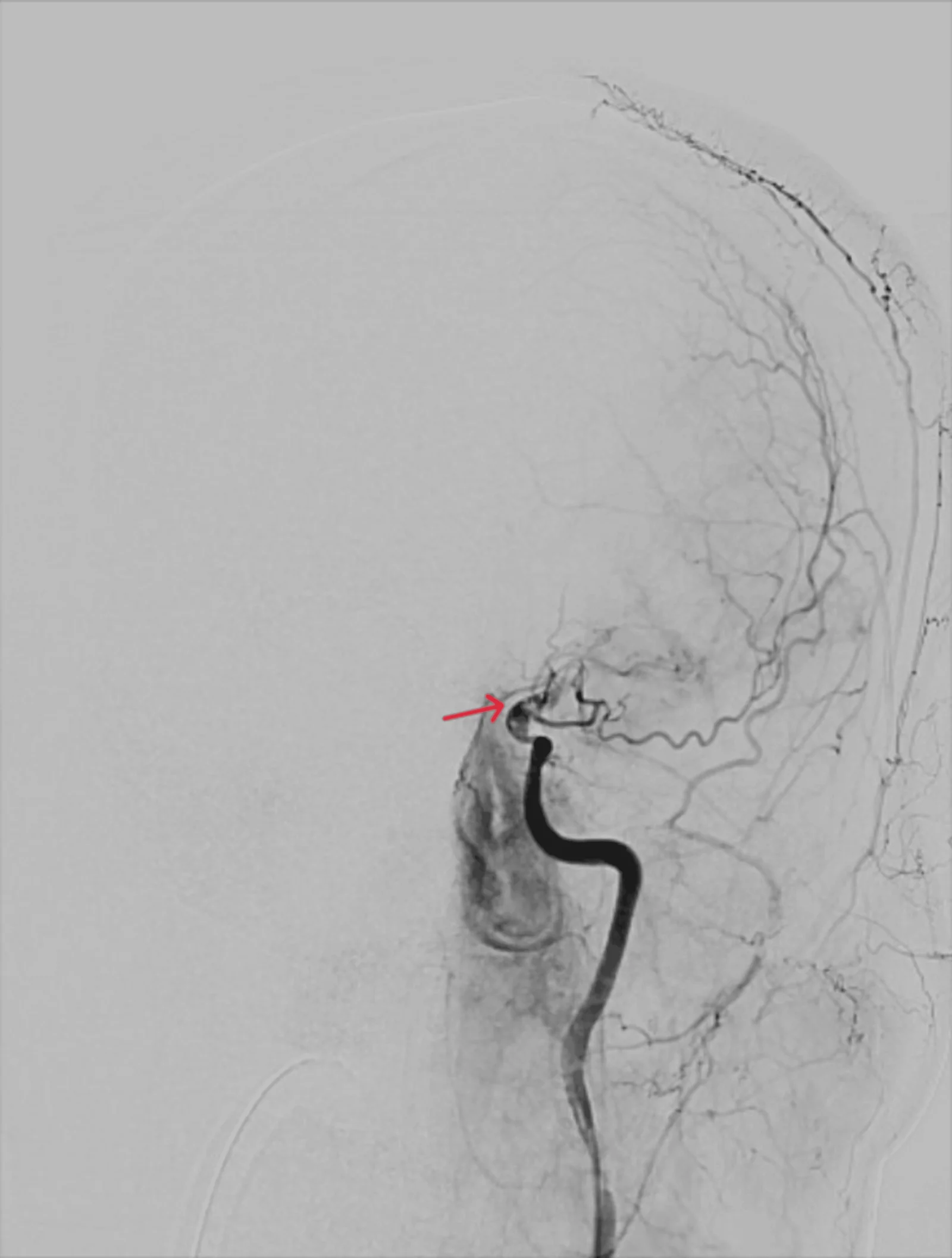
Digital subtraction angiography (DSA) of the head and neck demonstrating occlusion of the distal (terminal) segment of the left internal carotid artery (as indicated by red arrow).

**Figure 3:**
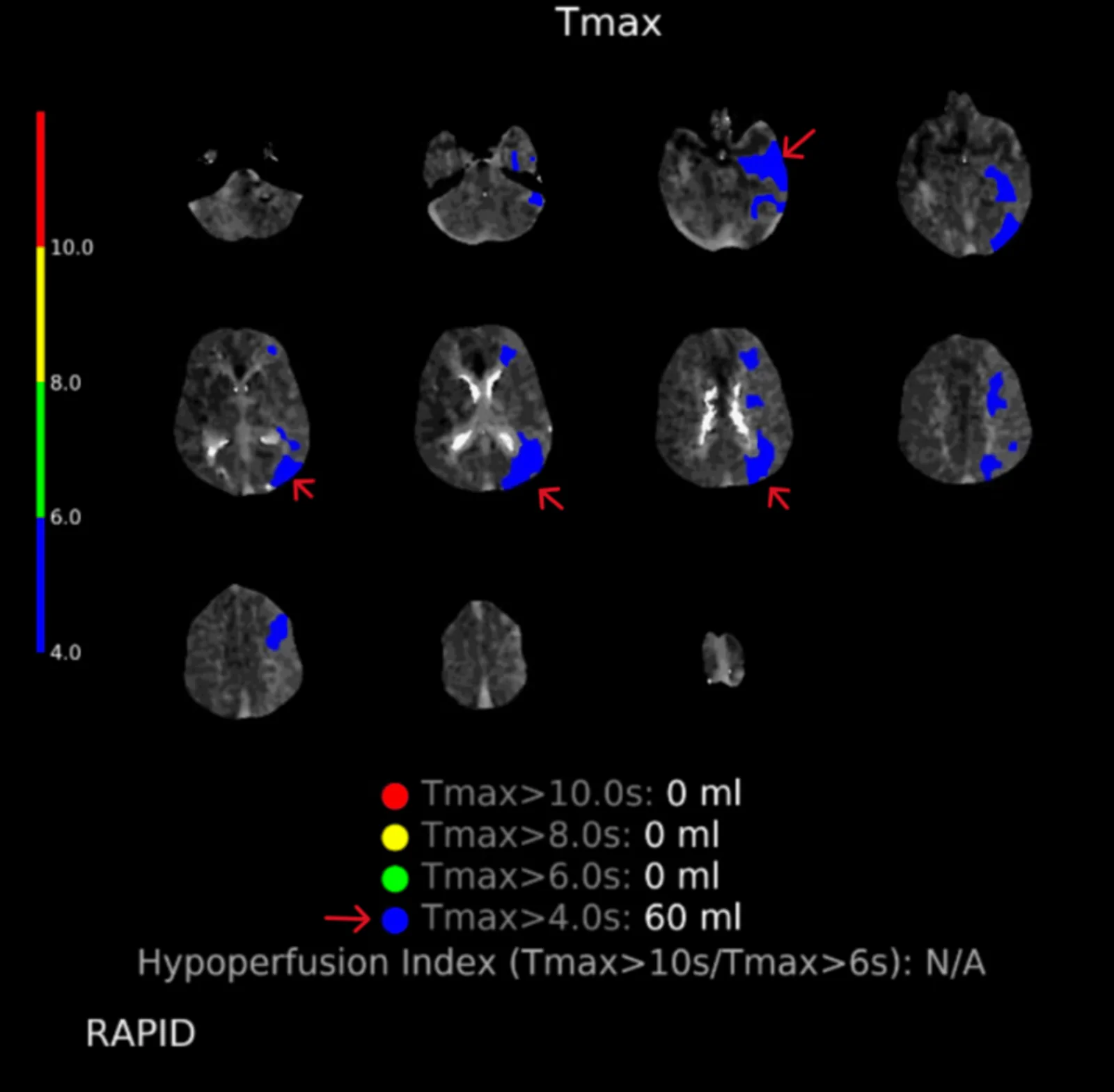
CT perfusion scans demonstrating prolonged contrast transit, with a time-to-maximum (Tmax) >4 seconds involving approximately 60 mL of the left cerebral hemisphere (as indicated by the red arrows).

**Figure 4:**
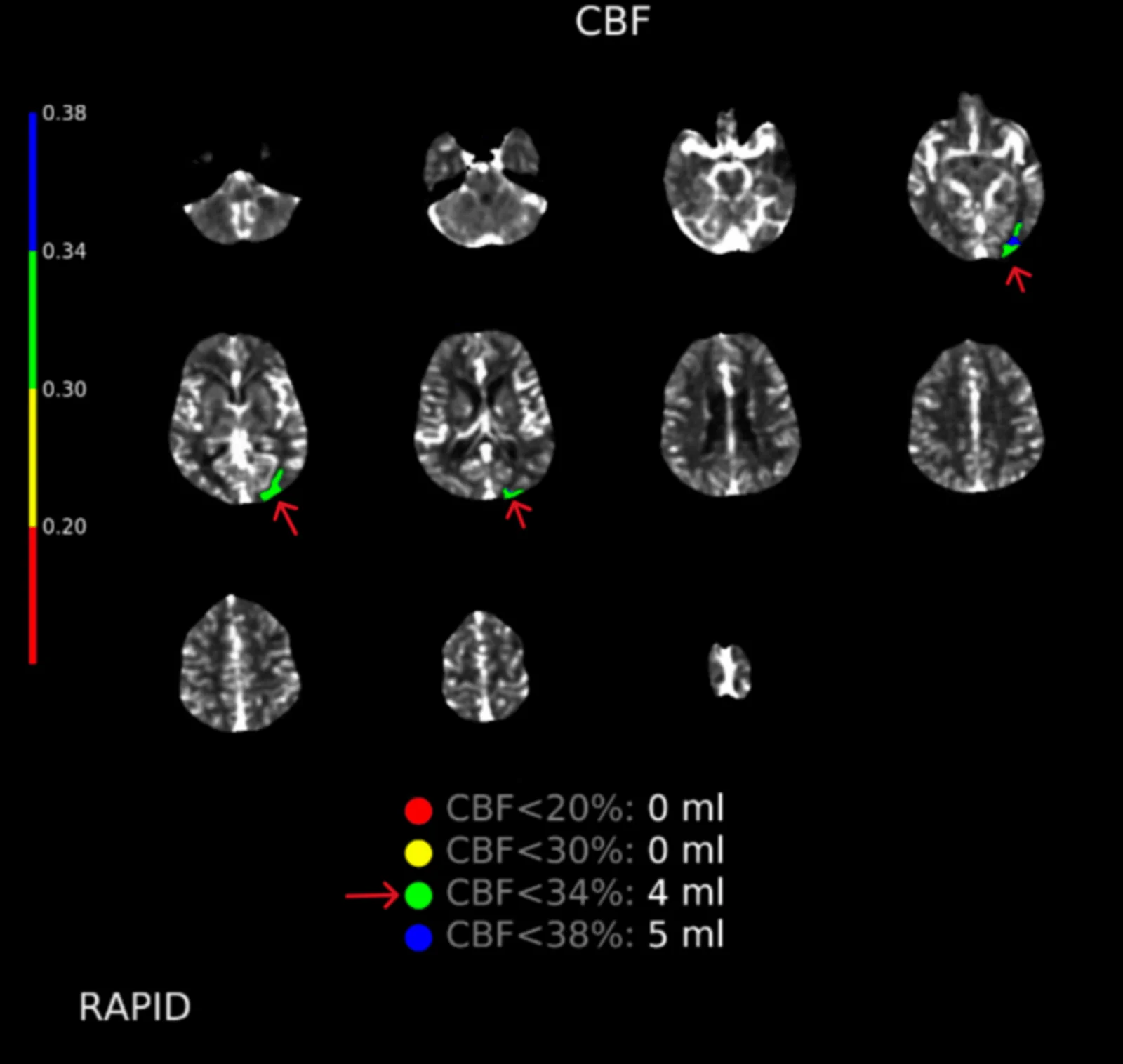
CT perfusion scans demonstrating a small region of reduced cerebral blood flow (CBF <34%), corresponding to an estimated ischemic core volume of 4 mL (as indicated by the red arrows).

Digital subtraction angiography (DSA) of the head and neck demonstrated complete occlusion of the distal (terminal) segment of the left ICA (Figure 2). CT perfusion imaging (Figures 3-4) demonstrated mild prolongation of contrast transit, with a time-to-maximum (Tmax) >4 seconds delay involving approximately 60 mL of the left cerebral hemisphere, predominantly within the middle cerebral artery (MCA) territory. The ischemic core was minimal, with a CBF volume of <34% measuring 4 mL. More stringent perfusion thresholds (CBF <30% and Tmax >6 seconds) demonstrated no established infarct core or clinically significant perfusion mismatch.

Acute management and reperfusion therapy decision

Despite the small ischemic core demonstrated on perfusion imaging, the patient was outside the therapeutic time window for intravenous thrombolysis (IVT) and was therefore initiated on aspirin 300 mg. Following multidisciplinary consensus, emergent endovascular thrombectomy was performed because of the terminal ICA occlusion, small ischemic core, delayed perfusion findings, and disabling cortical deficits.

Mechanical thrombectomy and procedural outcome

**Figure 5:**
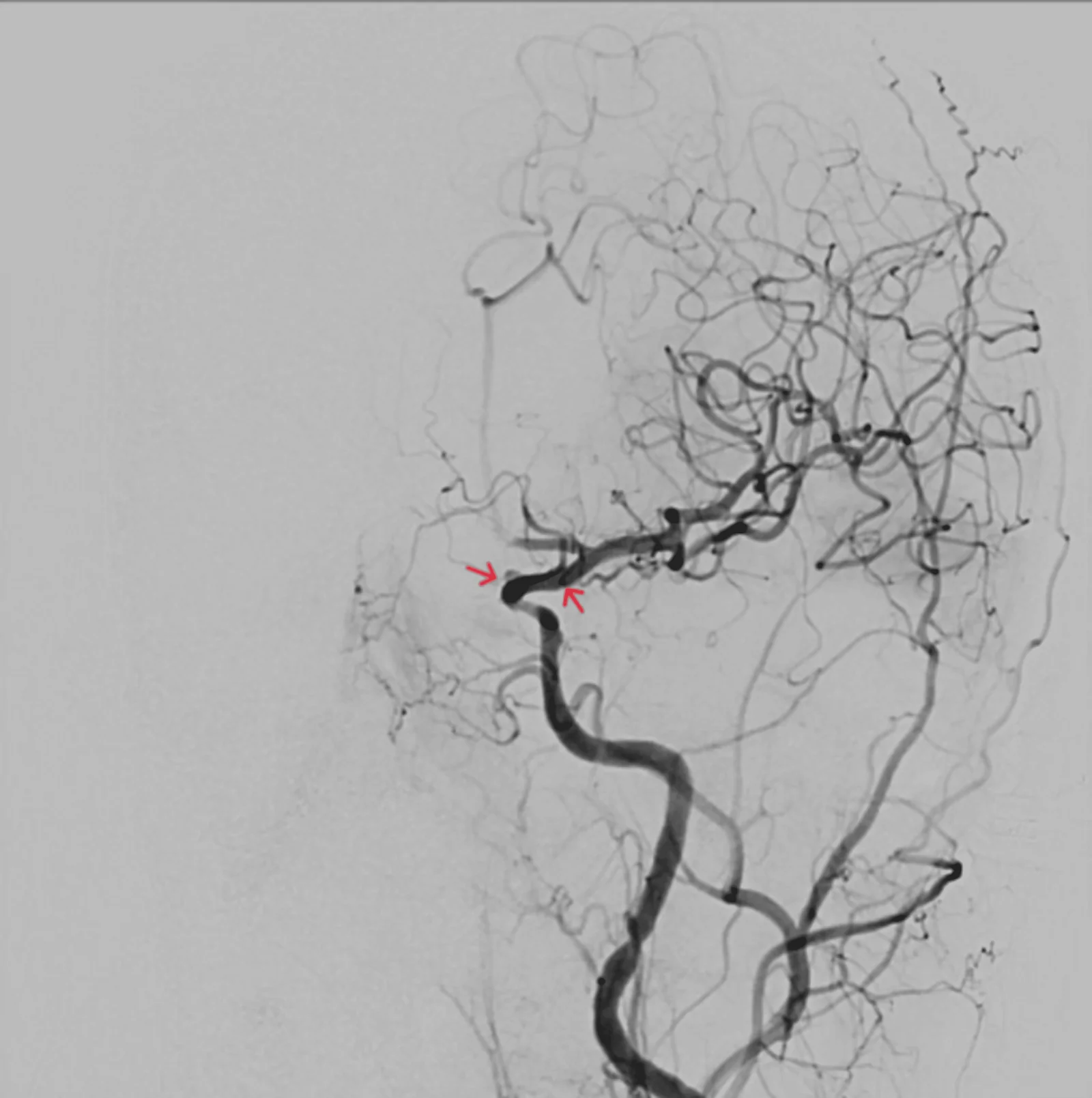
Digital subtraction angiography (DSA) following mechanical thrombectomy demonstrating complete reperfusion of the left terminal internal carotid artery with Thrombolysis in Cerebral Infarction (TICI) grade 3 flow (as indicated by the red arrows).

Successful mechanical thrombectomy of the left terminal ICA occlusion was performed, achieving complete reperfusion with a TICI grade of 3 (Figure 5). No stents were placed, and there were no immediate intraprocedural complications.

Post-procedural imaging and intensive care unit (ICU) course

**Figure 6:**
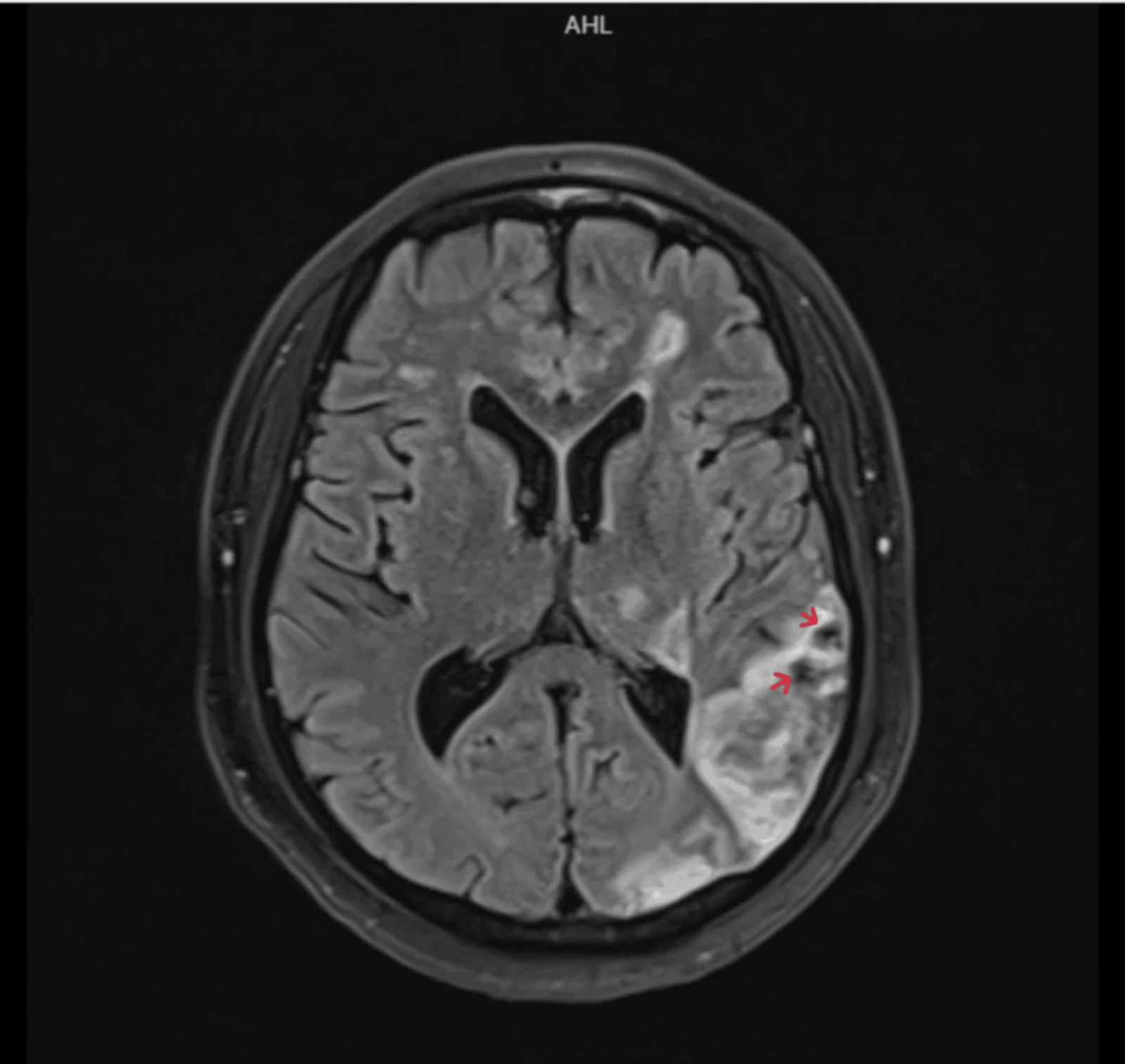
Post-thrombectomy MRI demonstrating acute left ICA territory infarction involving the hemisphere and thalamus, with evidence of focal hemorrhagic transformation in the parietal region (as indicated by red arrows). ICA: internal carotid artery

Post-procedure, the patient was admitted to the ICU for close monitoring. A follow-up brain magnetic resonance imaging (MRI) demonstrated recent infarction in the left cerebral hemisphere with evidence of hemorrhagic transformation (Figure 6). Follow-up CT imaging confirmed stability, and the patient was transitioned to aspirin 75 mg daily once imaging remained stable. The patient was subsequently transferred to the stroke unit.

Neurological evolution and hospital course

**Figure 7:**
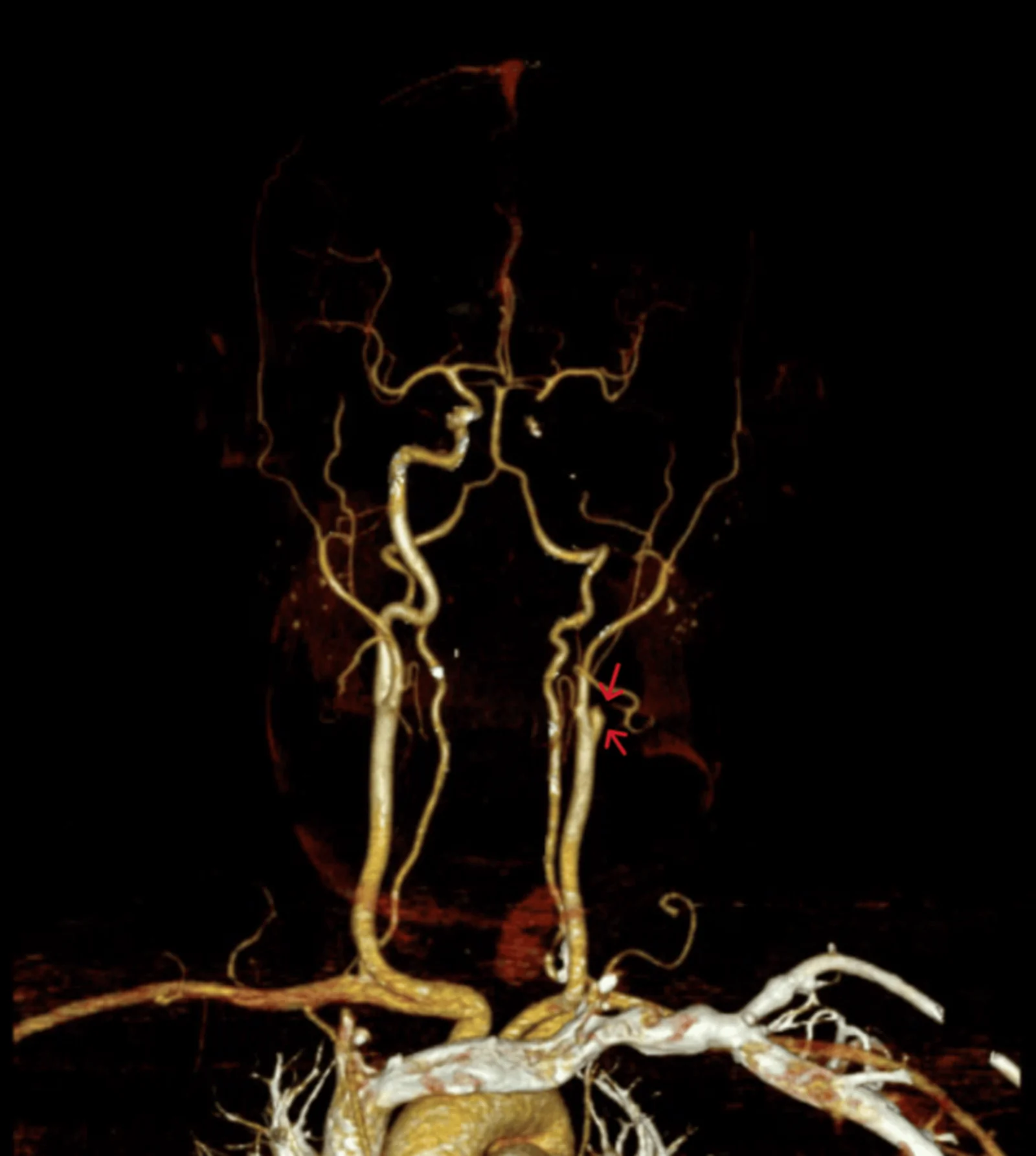
CT angiography (CTA) of the head and neck demonstrating complete occlusion of the left internal carotid artery from just distal to its origin, involving the cervical, petrous, cavernous, and terminal segments (as indicated by red arrows).

**Figure 8:**
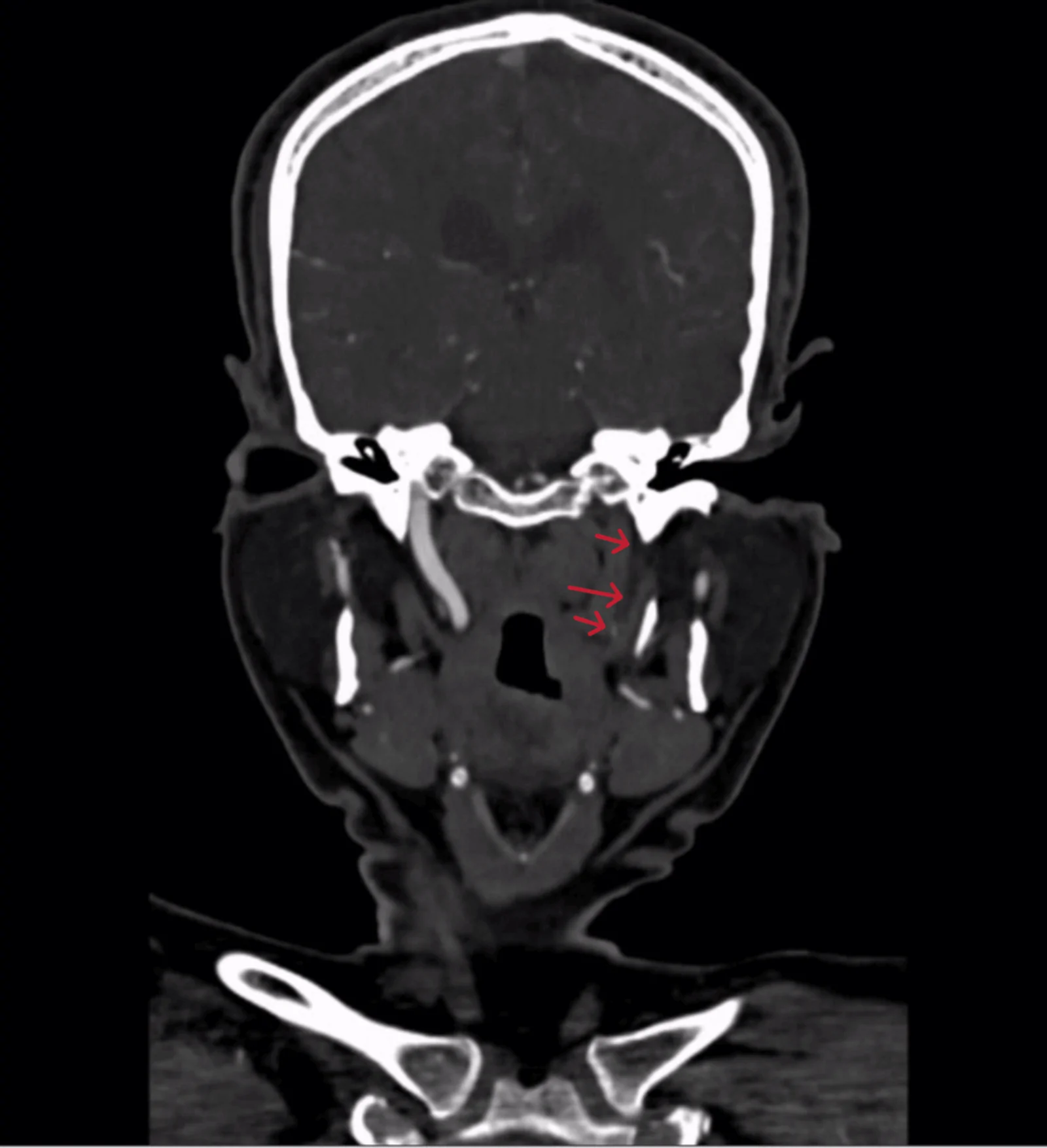
CT angiography (CTA) of the head and neck demonstrating filling defect of the left internal carotid artery (as indicated by red arrows).

During the first few days of hospitalization, the patient remained aphasic with significantly impaired comprehension but maintained full motor strength (5/5 bilaterally). His neurological status fluctuated, with NIHSS scores ranging from 6 to 7, primarily driven by the severity of the language deficit. Repeat vascular imaging revealed re-occlusion of the left ICA (Figures 7-8), which was managed conservatively as the patient remained clinically stable with good collateral circulation and no neurological deterioration.

Etiological workup

The etiological workup included cardiac monitoring, which showed no evidence of atrial fibrillation, and a transthoracic echocardiogram, which revealed no cardioembolic source. Extensive laboratory investigations were unremarkable except for the identification of poor glycemic control, for which insulin therapy was initiated.

Clinical recovery and discharge

By day 10, the patient showed gradual improvement in aphasia with supportive care and intensive speech therapy. He remained clinically stable on antiplatelet and statin therapy. At discharge, he was neurologically stable but had persistent sensory aphasia with preserved motor strength. His final NIHSS score was 7. He was discharged for repatriation to his home country with plans for continued long-term speech rehabilitation and secondary stroke prevention.

## Discussion

Acute ischemic stroke presenting primarily with confusion or disorientation remains a diagnostic challenge in the ED. Conventional stroke recognition tools such as BEFAST prioritize motor, speech, and visual deficits and do not explicitly assess higher cortical dysfunction such as impaired orientation or global cognitive disturbance [1,3]. In ED practice, BEFAST is an effective rapid triage tool but should not replace clinical judgment. Patients presenting with abrupt unexplained cognitive change should still be evaluated for acute ischemic stroke, even when an initial rapid bedside screening assessment does not identify obvious focal neurological deficits. Comprehensive neurological examination and timely neuroimaging may subsequently reveal cortical signs that were not appreciated during the initial evaluation. As a result, patients whose initial presentation is dominated by cognitive symptoms may be at increased risk of delayed stroke recognition and delayed access to reperfusion therapies [9].

Stroke code activation in this patient was prompted by abrupt unexplained confusion despite an initially unremarkable rapid bedside assessment, in which no obvious limb weakness, facial asymmetry, dysarthria, or gross visual deficits were appreciated. Formal neurological examination performed after neuroimaging subsequently demonstrated sensory aphasia, a right visual field deficit, and impaired finger-to-nose testing, resulting in an NIHSS score of 7. Rather than suggesting that cortical deficits were absent, this case illustrates that they were not appreciated during the initial rapid bedside assessment. This approach is consistent with current guideline recommendations emphasizing that clinical judgment should supersede rigid screening tools when evaluating patients with abrupt neurological change [1]. Acute confusion may represent an early manifestation of dominant hemisphere cortical ischemia in LVO and should prompt consideration of stroke even before more specific cortical deficits become clinically apparent [3,4,8].

The concept of “Stroke Chameleons” is particularly relevant in this context. Stroke chameleons describe ischemic strokes that present with atypical or non-focal features, including confusion, delirium, or isolated cognitive impairment, and are frequently misattributed to metabolic, toxic, or systemic causes [9]. Several ED-based studies have demonstrated that reliance on traditional stroke scales alone may fail to identify these presentations, reinforcing the importance of maintaining a high index of suspicion and pursuing early neuroimaging when acute neurological change is otherwise unexplained [9-11].

Once stroke is suspected, early multimodal imaging plays a pivotal role in guiding management. In our patient, CT angiography revealed a terminal ICA occlusion despite relatively preserved motor function. Although CT perfusion demonstrated a small ischemic core with a region of delayed contrast transit, multidisciplinary discussion favoured mechanical thrombectomy because of the terminal ICA occlusion and concern for ongoing thrombus propagation. Successful reperfusion (TICI 3) was achieved. This management approach is supported by five landmark thrombectomy trials demonstrating that mechanical thrombectomy significantly improves functional outcomes in anterior circulation LVO, independent of IV thrombolysis eligibility [4]. Patients with predominantly cortical deficits despite minimal motor impairment may still harbour LVO and benefit from timely reperfusion when appropriately selected [4,5].

Emerging literature further supports the need to broaden stroke recognition beyond traditional screening paradigms. A recent observational study demonstrated that patients with stroke chameleons exhibit distinct clinical and radiological characteristics compared with patients presenting with more typical ischemic stroke syndromes, highlighting the importance of recognizing atypical presentations in the ED [11]. Similarly, contemporary analyses emphasize that cortical symptoms alone should be considered potentially disabling, warranting urgent stroke pathway activation and vascular imaging [5,11]. These findings reinforce the rationale for stroke code activation in the present case, despite an initially unremarkable BEFAST assessment.

Re-occlusion following successful mechanical thrombectomy is an uncommon but recognized complication after endovascular treatment for acute ischemic stroke. Reported mechanisms include persistent thrombogenic pathology, procedure-related endothelial injury, and underlying large artery atherosclerosis. In our patient, repeat intervention was not pursued because neurological examination remained stable, collateral circulation was preserved, and there was no evidence of clinical deterioration. This highlights the importance of individualized management following successful reperfusion [12].

Finally, this case highlights the dynamic nature of LVO stroke, illustrated by subsequent ICA re-occlusion managed conservatively due to preserved collateral flow and clinical stability. Such evolution underscores the importance of ongoing neurological assessment and individualized management, even after successful reperfusion. Early recognition of predominantly cognitive or cortical presentations, rapid imaging, and timely intervention with mechanical thrombectomy contributed to a favourable neurological outcome in this patient. As a single-case report, our findings cannot be generalized to all patients with atypical stroke presentations. Nevertheless, this case highlights an important diagnostic challenge and reinforces the value of maintaining a high index of suspicion for acute ischemic stroke in patients presenting with abrupt unexplained cognitive symptoms.

## Conclusions

This case highlights the importance of recognizing acute confusion and disorientation as potential presenting features of ischemic stroke, particularly in the ED setting. A normal initial bedside stroke screen does not exclude LVO. Persistent unexplained confusion warrants repeated neurological assessment and consideration of advanced neurovascular imaging. Early stroke code activation based on clinical judgment enabled prompt neuroimaging, identification of a terminal ICA occlusion, and timely reperfusion therapy in this patient. Our findings reinforce the need for heightened clinical vigilance when evaluating acute cognitive changes and support a broader approach to stroke recognition that prioritizes symptom acuity over classical deficit patterns.
